# Correlation between a real-time bioparticle detection device and a traditional microbiological active air sampler monitoring air quality in an operating room during elective arthroplasty surgery: a prospective feasibility study

**DOI:** 10.2340/17453674.2025.43002

**Published:** 2025-02-24

**Authors:** Lise-Lott LARSSON, Johan NORDENADLER, Gunilla BJÖRLING, Li FELLÄNDER-TSAI, Stergios LAZARINIS, Bengt LJUNGQVIST, Janet MATTSSON, Berit REINMÜLLER, Harald BRISMAR

**Affiliations:** 1 Division of Orthopaedics and Biotechnology, CLINTEC, Karolinska Institutet, Stockholm, Sweden; 2 Department of Neurobiology, Care Sciences and Society, Karolinska Institutet, Stockholm, Sweden; 3 Jönköping University, School of Health and Welfare, Jönköping. Sweden; 4 Department of Reconstructive Orthopedics, Karolinska University Hospital, Stockholm, Sweden; 5 Department of Surgical Sciences/Orthopaedics, Uppsala University, Sweden; 6 Department of Architecture and Civil Engineering, Division of Building Services Engineering, Chalmers University of Technology, Göteborg, Sweden; 7 University of Southeast Norway, Sörost, Norway

## Abstract

**Background and purpose:**

The standard method for controlling operating room (OR) air quality is measuring bacteria-carrying particles per volume unit of air: colony forming units (CFU/m^3^). The result takes at least 2 days after sampling. Another method is real-time measurements of fluorescing bioparticles per unit volume of air (FBP/dm^3^). We aimed to compare simultaneous measurements of FBP/50 dm^3^ and CFU/m^3^ during ongoing arthroplasty surgery.

**Methods:**

18 arthroplasties were performed in a modern OR with turbulent mixed airflow ventilation. The sampling heads of a BioAerosol Monitoring System (BAMS) and a microbiological active air sampler (Sartorius MD8 Air Sampler) were placed next to each other, and 6 parallel 10-minute registrations of FBP/50 dm^3^ and CFU/m^3^ were performed for each surgery. Parallel measurements were plotted against each other, Passing–Bablok nonparametric linear regression was performed, and the Spearman correlation coefficient (r) was calculated.

**Results:**

The *r* between FBP ≥ 3 μm/50 dm^3^ and CFU/m^3^ sampled for 96 x 10-minute intervals, was 0.70 (95% confidence interval [CI] 0.57–0.79). In the 25th percentile with the lowest 10-minute FBP ≥ 3μm/50 dm^3^, there were no CFU measurements with ≥ 10 and 4% with ≥ 5 CFU/m^3^. In the 75th percentile with the highest 10-minute FBP ≥ 3 μm/50 dm^3^, there were 58% CFU measurements with ≥ 10 and 88% with ≥ 5 CFU/m^3^. The *r* between FBP ≥ 3 μm/50 dm^3^ and CFU/m^3^ means sampled during 18 operations was 0.87 (CI 0.68–0.95).

**Conclusion:**

Low FBP ≥ 3 μm/50 dm^3^ measured by BAMS indicates low CFU/m^3^; conversely, high FBP ≥ 3 μm/50 dm^3^ indicates high CFU/m^3^. Real-time measurements of FBP ≥ 3 μm/50 dm^3^ can be used as a supplement to CFU/m^3^ monitoring OR air bacterial load.

A periprosthetic joint infection (PJI) affects about 1–2% of all patients undergoing total hip or total knee arthroplasty [1-3] and comes with substantial morbidity and costs [4-5]. The importance of ultraclean operating room (OR) air for preventing PJI was already demonstrated in 1969 by Charnley [6] and in the 1980s by Lidwell et al. [7].

Airborne particles released from the surgical team, or the patient may carry microorganisms that settle into the wound or contaminate implants and instruments [8]. The OR ventilation system is designed to prevent this by HEPA-filtered air either through a unidirectional airflow (UDAF) ventilation system or a turbulent mixed airflow (TMA) ventilation system [9]. Factors that have an impact on the level of microorganisms in the air are the number of healthcare professionals present in the OR [10,11], type of clothing systems [12], healthcare professionals’ activity level [13], and door openings [14,15].

The gold standard for monitoring OR air quality is the analysis of bacteria aerobic colony forming units per 1 m^3^ air (CFU/m^3^) [9]. The preferred method of measuring CFU/m^3^ is using a volumetric active air sampler with the sampling head 30 cm from the surgical area. The process is rather laborious, and the results are not available earlier than 2 days after sampling. Thus, it is not convenient for continuous real-time OR-air surveillance and is difficult to use for studies on time-dependent events during surgery. An alternative method could be measuring airborne fluorescent bioparticles (FBP) in real time. As the intended use is to have a system always running and not disturbing the surgery workflow, the FBP sampling head is positioned 2–3 meters from the surgical area.

We aimed to evaluate how simultaneous FBP/50 dm^3^ and CFU/m^3^ measurements correlate during live elective arthroplasty.

## Methods

### Study design

This prospective noninterventional feasibility study compares 2 methods of measuring OR air quality during arthroplasty surgery: the reference standard CFU/m^3^ and the index method FBP/50 dm^3^.

The study was reported according to Strobe guidelines.

### Settings

**Figure 1:**
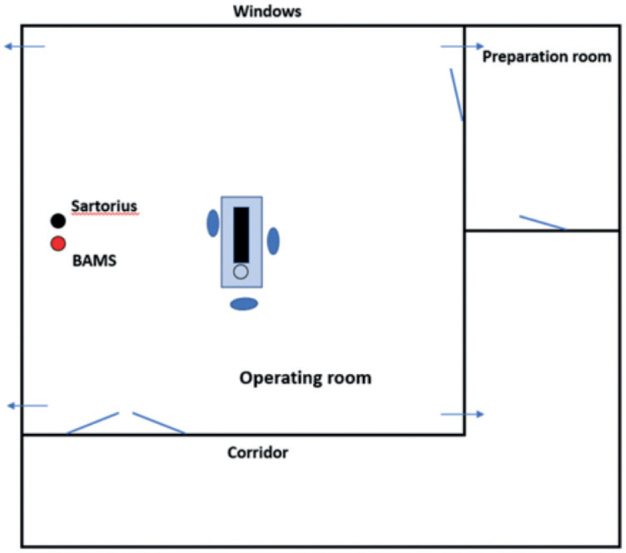
Outline of the TMA ventilated operating room. The Sartorius MD8 and the BAMS sampling heads (black and red dot) were positioned 1 m apart and 3.2 m from the surgery area, 1.2 m above the floor. Arrows indicate exhaust air devices. Sartorius = aerobic CFU/m^3^ measurement device. BAMS = BioAerosol Monitoring System, which measures fluorescing bioparticles (FBP/dm^3^).

The study was conducted from November 2022 to January 2023 during 18 arthroplasties in one OR (57.6 m^2^) at the Karolinska University Hospital, Stockholm. Surgeries included were elective arthroplasties scheduled on the dates when both measuring devices were available. The OR was equipped with a TMA ventilation system with an airflow of 2,600 L/s and 48.8 air changes per hour. 24 air supply diffusers, each 60 x 60 cm with HEPA filter, were in the ceiling above the center of the room. 4 exhaust air devices were in the corners (23 cm above the floor), and another 4 are placed 30 cm from the ceiling (Figure 1). All surgical staff members wore disposable 100% polypropylene surgical clothes (Clean Air Suit, Mölnlycke Health Care AB, Gothenburg, Sweden).

### Measuring devices

Aerobic CFU/m^3^ measurements were conducted using a traditional air sampler, Sartorius MD8 (Sartorius AG, Göttingen, Germany), where 1 m^3^ of air was drawn through a gelatine filter for 10 minutes. The filters (collection capacity ≥ 3 μm) were placed on blood agar plates (90 mm diameter Petri dishes) and incubated for 48 hours (aerobe, 35°C), and the numbers of aerobic CFU/m^3^ were counted. Plates contaminated during the sampling process and plates with condensation under the lid at analysis were discarded.

Fluorescing bioparticles were counted using a real-time measuring device, Bio Aerosol Monitoring System (BAMS, Zecon AB, Stockholm, Sweden), where 5 dm^3^ air per minute was drawn into the measuring device and illuminated with a laser beam for 5-second periods. Illuminated particles scatter light depending on size and number, and if they contain nicotinamide adenine dinucleotide + hydrogen (NADH) or riboflavin (indicating biological activity), they fluorescence. Particle sizes 0.5–< 1, 1–< 2, 2–< 3, 3–< 5, 5–< 10, and ≥ 10 μm are counted.

The number of particles counted during 120 x 5-second periods (10 minutes) was registered as number FBP/50 dm^3^ to have a measurement comparable to the 10-minute CFU/m^3^ sampling.

As the pore size in the detection filter of the volumetric air sampler, Sartorius MD8, is ≥ 3 μm, we have focused on particles ≥ 3 μm when comparing the instruments.

2 alternative FBP/50 dm^3^ registrations were saved. The first included all 10-minute periods of registered FBP/50 dm^3^. The second, introduced post hoc, excluded 10-minute periods with distinctly different patterns of registered particles (Table 1). These periods of supposed FBP measurement distortion were defined as 10-minute periods with ≥ 12 continuous, 5-second counts of FBPs 0.5–< 1 μm ≥ 10, whereof ≥ 1 5-second count was ≥ 20, and during the same period ≥ 1 5-second count of FBPs 3–< 5 μm was ≥ 3, and ≥ 1 5-second count of FBPs 5–< 10 μm was ≥ 2 (Table 2).

**Table 1:** Output data from BAMS on numbers and sizes of fluorescent bio particles (FBP) registered during consecutive 5-second periods

Time	Number of counted FBP of different sizes in μm per 5 s					
	0.5–< 1	1–< 2	2–< 3	3–< 5	5–< 10	≥ 10
Measurements when using diathermy						
15:18:33	34	19	8	3	0	0
15:18:38	68	31	13	3	0	0
15:1 8:43	73	34	13	2	0	0
15:1 8:48	111	49	11	1	0	1
15:18:53	1 00	47	20	6	0	0
15:18:58	92	36	11	3	0	0
15:19:03	1 04	48	17	3	0	0
15:19:08	1 09	41	11	5	3	0
15:19:13	84	34	13	2	3	0
15:19:18	76	33	7	4	0	0
15:19:23	87	34	8	3	2	0
15:19:28	77	30	11	3	0	0
15:19:33	86	30	13	2	2	0
15:19:38	60	29	9	0	0	0
15:1 9:43	63	21	5	2	0	0
15:1 9:48	56	28	5	4	1	0
Measurements without diathermy						
15:42:48	1	1	0	0	0	0
15:42:53	0	0	0	0	0	0
15:42:58	0	0	0	0	0	0
15:43:03	1	1	0	0	0	0
15:43:08	0	0	1	1	0	0
15:43:13	0	0	0	0	0	0
15:43:18	0	0	0	0	0	0
15:43:23	0	0	0	1	0	0
15:43:28	2	1	0	0	0	0
15:43:33	0	0	0	0	1	1
15:43:38	0	0	0	0	0	0
15:43:43	0	0	0	0	1	0
15:43:48	0	0	0	1	0	0
15:43:53	0	0	1	0	0	0
15:43:58	0	0	0	0	0	0
15:44:03	0	0	0	0	0	0

Note in measurements when using diathermy 1 minute of continuous 5-second periods with high counts of small particles (size 0.5 μm), and at the same time high counts of larger particles (3μm and 5μm) when intensive diathermy was used. In measurements without diathermy a more normal pattern is seen without intensive diathermy disturbance. Time is the registration time in hh:mm:ss.

**Table 2:** Definition of periods with FBP disturbance: all requirements need to be fulfilled

FBP size (μm)	Exclusion requirement
0.5–< 1	1-minute continuous 5 s counts ≥ 10 whereof ≥ 1 count ≥ 20
3–< 5	≥ 1 x 5 s count ≥ 3 during the 1 minute of FBP 0.5–< 1 ≥ 10
5–< 10	≥ 1 x 5 s count ≥ 2 during the 1 minute of FBP 0.5–< 1 ≥ 10

### Data collection

The Sartorius MD8 and the BAMS sampling heads were positioned 1 m apart and 3.2 m from the surgery area, 1.2 m above the floor (Figure 1). Parallel measurements started directly after the skin incision and continued for 6 x 10-minute periods, with 5 x 2–3-minute break intervals for the Sartorius MD8 filter change. The sample size was chosen to give about 100 paired samples, which was estimated to be sufficient compared with previous similar studies [16]. No data on patient identity was saved.

### Statistics

Spearman’s rank correlation coefficients (r) with 95% confidence intervals (CI) were calculated comparing FBP ≥ 3 μm/50 dm^3^ and CFU/m^3^ for 10-minute periods and mean FBP ≥ 3 μm/50 dm^3^ and mean CFU/m^3^ for 60-minute surgery periods. Passing–Bablok regression was applied to estimate the correlation between the FBP ≥ 3 μm/50 dm^3^ and CFU/m^3^ measurements. The method results in a nonparametric linear regression, taking care of the measurement errors and outliers. Data was analyzed in SPSS version 29 (IBM Corp, Armonk, NY, USA) and MedCalc version 23.0.6 - 32 bit (MedCalc Software, Ostend, Belgium).

### Ethics, data sharing, funding, and disclosures

Because of the study design, including no personal data processing and no patient intervention, the Swedish Ethical Review Authority found the study exempt from the Swedish Ethical Review Act and did not consider a formal ethical review relevant (Swedish Ethical Review Authority, 2022-03554-01). The data in this study is presented in Supplementary data. LÖF, the Swedish patient insurance, supported the study financially. The authors declare no conflict of interest. Complete disclosure of interest forms according to ICMJE are available on the article page, doi: 10.2340/17453674.2025.43002

## Results

**Figure 2:**
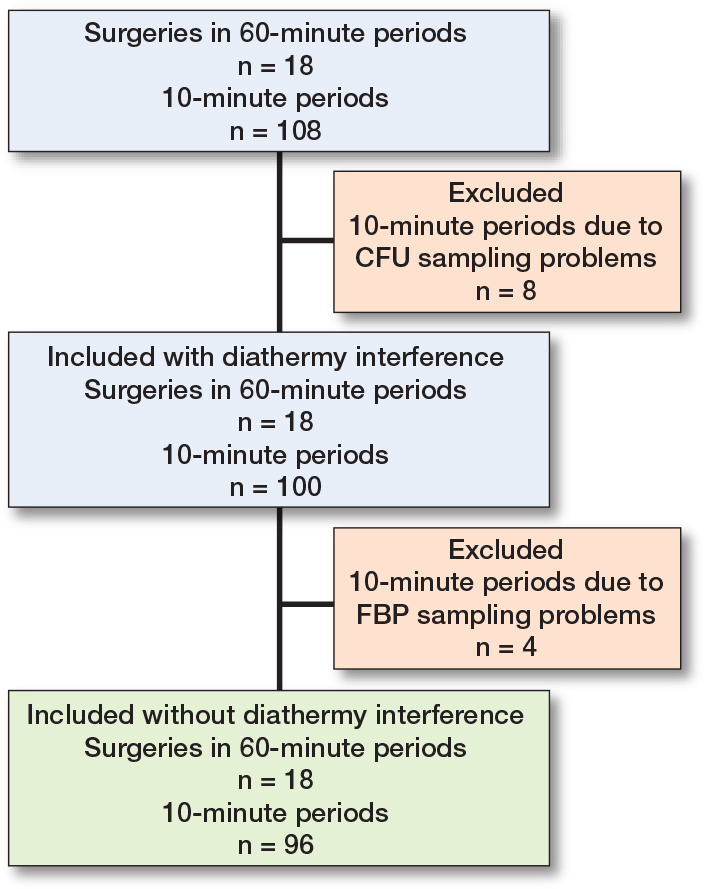
Flowchart of included 10-minute and mean surgery CFU/m^3^ and FBP/50 dm^3^ registrations with and without diathermy interference. CFU sampling problems occurred in 3 x 10-minute periods in 1 surgery, in 2 x 10-minute periods in 1 surgery, and in 1 x 10-minute period in 2 surgeries. Diathermy interference occurred in 2 x 10-minute periods in 1 surgery and in 1 x 10-minute period in 2 surgeries.

**Figure 3:**
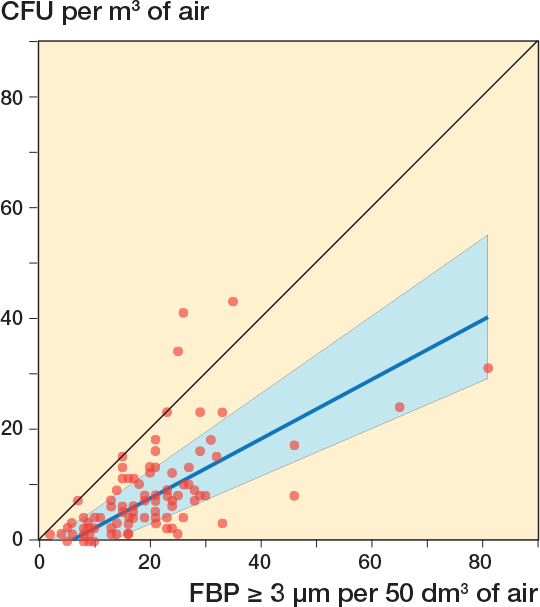
Correlation between 96 simultaneous 10-minute registrations of FBP ≥ 3 μm/50 dm^3^ (6 x 10-minute periods/surgery) and CFU/m^3^ (6 x 10-minute periods/surgery) during 18 arthroplasties. The blue line represents the regression line (Passing–Bablok regression; y = –3.31 + 0.54x). The blue field represents the 95% confidence interval. 4 x 10-minute periods with diathermy interference have been excluded. Spearman’s rank correlation coefficient, *r* = 0.70 (CI 0.57–0.79).

**Figure 4:**
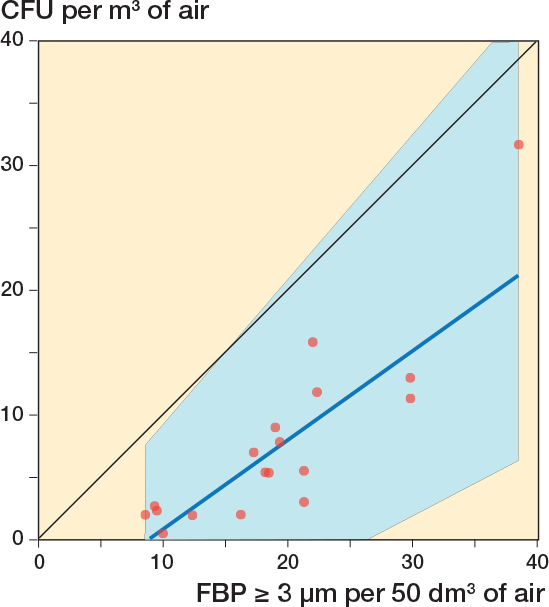
Correlation between simultaneous 60-minute registrations of FBP ≥ 3 μm/50 dm^3^ (average of 6 x 10-minute periods) and CFU/m^3^ (average of 6 x 10-minute periods) during 18 arthroplasties. 4 x 10-minute periods (2 in 1 surgery and 1 in 2 surgeries) with diathermy interference have been excluded. The blue line represents the regression line (Passing–Bablok regression; y = –6.37 + 0.72x). The blue field represents the 95% confidence interval. Spearman’s rank correlation coefficient, *r* = 0.87 (CI 0.68–0.95).

18 surgeries were included, containing 108 x 10-minute periods. Due to a technical failure of the CFU analysis, 8 of 108 agar plates were discarded (Figure 2). 4 x 10-minute periods contained 1-minute periods meeting the post hoc formulated definition of FBP measurement distortion (Figure 2). If these periods were excluded, the *r* between FBP ≥ 3 μm/50 dm^3^ and CFU/m^3^ for the remaining 96 parallel 10-minute intervals was 0.70 (CI 0.57–0.79) (Figure 3) and for the 18 operation periods 0.87 (CI 0.68–0.95) (Figure 4). Periods of FBP distortion coincided with extensive use of diathermy and the smell of smoke in the OR.

In the 25th percentile, with the lowest 10-minute FBP ≥ 3 μm/50 dm^3^, there were no CFU measurement results with ≥ 10 CFU/m^3^ and 4% with ≥ 5 CFU/m^3^. In the 75th percentile with the highest 10-minute FBP ≥ 3 μm/50 dm^3^, 88% had CFU/m^3^ measurements with ≥ 5 and 58% ≥ 10 CFU/m^3^.

**Figure 5:**
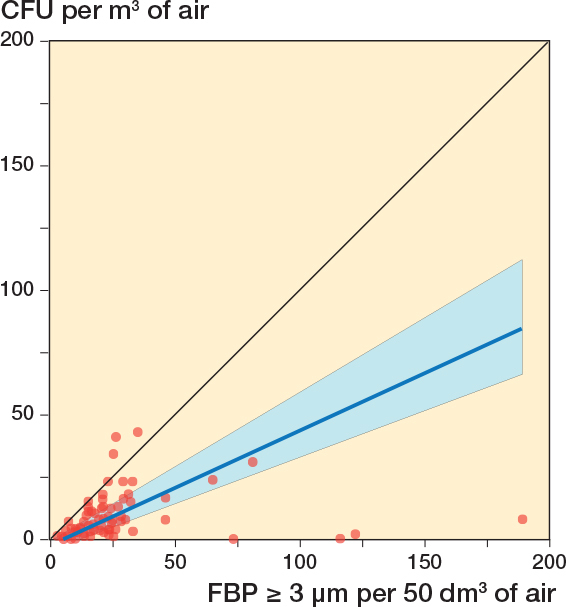
Correlation between 100 simultaneous 10-minute registrations of FBP ≥ 3 μm/50 dm^3^ (6 x 10-minute periods/surgery) and CFU/m^3^ (6 x 10-minute periods/surgery) during 18 arthroplasties. Diathermy interference has not been adjusted for. The blue line represents the regression line (Passing–Bablok regression; y = –2.39 + 0.46x). The blue field represents the 95% confidence interval. Spearman’s rank correlation coefficient, *r* = 0.57 (CI 0.41–0.69).

**Figure 6:**
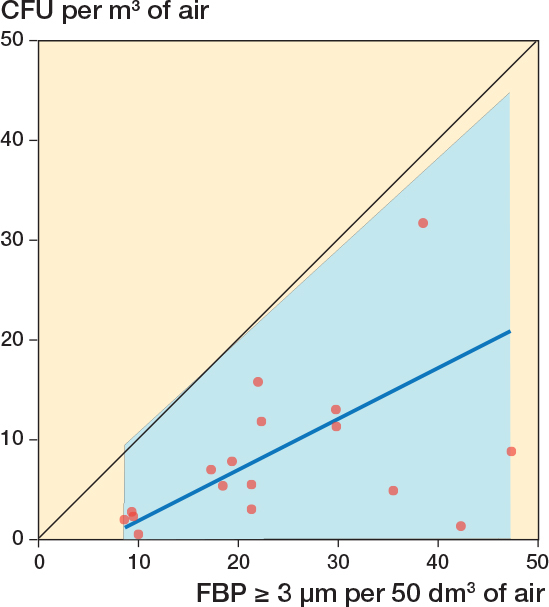
Correlation between simultaneous 60-minute registrations of FBP ≥ 3 μm/50 dm^3^ (average of 6 x 10-minute periods) and CFU/m^3^ (average of 6 x 10-minute periods) during 18 arthroplasties. Diathermy interference has not been adjusted for. The blue line represents the regression line (Passing–Bablok regression; y = –3.21 + 0.51x). The blue field represents the 95% confidence interval. Spearman’s rank correlation coefficient, *r* = 0.54 (CI 0.09–0.81).

If the 4 x 10-minute periods with FBP measurement distortion were not excluded the correlation for 10-minute intervals was 0.57 (CI 0.41–0.69) (Figure 5) and for surgeries 0.54 (0.09–0.81) (Figure 6).

## Discussion

We aimed to compare simultaneous FBP/50 dm^3^ and CFU/m^3^ measurements during ongoing arthroplasty surgery. FBP ≥ 3 μm/50 dm^3^ correlated with CFU/m^3^ values only if adjustment was made for FBP distortion.

A good correlation between FBP and CFU has been reported in a study by Dai et al. [17], even though that study had a smaller sample than ours, included OR periods before and after surgery, and did not distinguish different sizes of fluorescent bioparticles. A controlled test chamber study recently showed a moderate correlation between FBP and CFU [18]. There are studies where no correlation has been found [19,20]. These studies used other models of FBP instruments, and they were performed during other conditions, e.g., lower airflow, more permeable clothing systems, analyzing particles of different sizes, and not adjusting for diathermy interference. Studies on the correlation between airborne particles and CFU, not discriminating between inert particles and fluorescent bioparticles, have been conflicting [11,21,22]. Obviously, non-bioactive particles are not detected when counting CFU. Simultaneous detection of numbers of particles and bioactivity, therefore, enhances the specificity of airborne microbiological particle measurements.

Real-time FBP/50 dm^3^ monitoring has several advantages compared with conventional CFU/m^3^ active air sampling. It enables continuous surveillance of OR air quality and could act as an early warning system if the ventilation system is not working properly. Real-time monitoring could also help implement OR infection prevention strategies such as limiting physical activity, number of OR personnel, door openings, clothing regimes, etc., by giving instantaneous feedback on air cleanliness to OR staff. A higher surgical staff awareness of PJI prevention and adherence to OR behavioral routines will lead to fewer OR-related PJIs and potentially reduce the enormous cost of hospital-acquired infections associated with implant surgery. Measurements of CFU/m^3^ cannot be replaced by FBP/50 dm^3^ to establish whether an OR complies with the national standard of OR air cleanliness; it is a supplement. CFU/m^3^ measurements should be used for setting the standard, while FBP/50 dm^3^ can be used to gain an indication of how the OR air bacterial load varies over time, where live FBP/50 dm^3^ levels are compared with historic FBP/50 dm^3^ levels of that specific OR. Even though the FBP/50 dm^3^ bacterial load estimates are not exact, they give an idea of whether the air quality of the OR is better or worse under the present condition.

### Strengths

We used a systematic evaluation of a new real-time OR air microbial detection device, correlating the result to the gold standard, a traditional volumetric air sampler.

### Limitations

First, excluding 4 x 10-minute periods with supposed diathermy interference resulting in FBP measurement distortion is an obvious shortcoming in this study and the bioparticle measuring method. Our definition of diathermy interference has not been validated and was constructed post hoc by analyzing patterns on multiple sequences of bioparticle registrations. Increased FBP related to diathermy has previously been described, and the theory is that biological tissue becomes vaporized and subsequently detected [17,23]. Small particles seem to be most easily affected, which can be accounted for by only registering particles ≥ 3 μm. The situation when diathermy also affects larger particles can be solved using an algorithm like the one used in our study. We acknowledge that particles < 3 μm also can carry bacteria. Not measuring those may underestimate the bacterial load. However, it is important to understand that both methods deliver estimates of air bacterial load and not the exact number of airborne bacterial particles. CFU may underestimate the numbers as some bacteria do not grow on the bacterial media used for CFU measurements, some bacteria need other settings to grow, and several bacteria can be the source of 1 colony. Conversely, bio-fluorescent particle counting may overestimate the numbers by counting dead bacteria, living cells that are not bacteria, and other auto-fluorescing materials.

Second, we positioned the measuring devices 3 meters from the surgical field and between the 2 doors entering the OR (Figure 1). Thus, the actual FBP/dm^3^ and CFU/m^3^ at the surgical site have not been measured and could differ from that measured at a 3-meter distance. The unusually high CFU/m^3^ levels found at some surgeries are likely explained by this. Anyhow, the air in modern TMA-ventilated ORs is effectively diluted and evenly distributed [9], meaning that the aerobic CFU/m^3^ and FBP/50 dm^3^ levels registered in different places mirror the total OR air cleanliness. Further studies and development of the BAMS technique to allow monitoring closer to the wound are warranted.

Moreover, we had to discard 8 CFU gelatin filters (8 x 10-minute periods) due to technical problems (condensation and contamination). The decision not to analyze these filters was taken before the correlation analysis. We do not believe this influenced the result except for decreasing the sample size. Lastly, we included only arthroplasties performed in an OR with TMA ventilation. Therefore, the result cannot be directly generalized to other types of surgery, even though a relationship between FBP and CFU would probably still be found but following another equation. The correlation between the 2 instruments in an OR with laminar airflow remains to be investigated.

### Conclusion

We found a correlation between FBP ≥ 3 μm/50 dm^3^ and CFU/m^3^ during arthroplasties in an OR with TMA ventilation. Low FBP ≥ 3 μm/50 dm^3^ measured by BAMS indicates low CFU/m^3^; conversely, high FBP ≥ 3 μm/50 dm^3^ indicates high CFU/m^3^.

*In perspective,* real-time measurements of FBP ≥ 3 μm/50 dm^3^ could be used as a supplement to CFU/m^3^ monitoring OR air bacterial load, provided adjustment is made for FBP distortion periods. It could act as an early warning system of high OR air bacterial load, but there is a need for a better understanding of the technology and how it could be used during live surgery.

## Supplementary Material



## Supplementary data

List of measurements are available as Supplementary data on the article homepage, doi: 10.2340/17453674.2025.43002

## Notes

HB, JN, LL: conception, study design, acquisition, analysis and interpretation of data, manuscript draft, manuscript editing and review. GB, JM: conception, manuscript editing and review. LF, SL: conception, analysis and interpretation of data, manuscript editing and review. BL, BR: conception, study design, analysis and interpretation of data, manuscript editing and review. 

Handling co-editors: Bart A Swierstra and Jonas Ranstam

*Acta* thanks Roberto Traversari and other anonymous reviewers for help with peer review of this manuscript.
