# Supplementary material for: Correlation between a real-time bioparticle detection device and a traditional microbiological active air sampler monitoring air quality in an operating room during elective arthroplasty surgery: a prospective feasibility study
Source: Acta Orthop. 2025 Feb 24;96:176–81. doi: 10.2340/17453674.2025.43002 (PMC11849163; doi:10.2340/17453674.2025.43002)
Supplement: Supplementary file 1 [file ActaO-96-43002-s1.pdf]

	Aerobic CFU	Viable particles/dm³ ≥0.5–<1 µm	1–<2 µm	2–<3 µm	3–<5 µm	5–<10 µm	≥10 µm	≥3 µm	≥5 µm	Ω Excluded 10 minutes periods
1:1		10	7	10	3	10	1	14	11	Excluded no CFU
1:2	0	1,458	325	40	7	1	2	10	3	
1:3	0	26	7	13	4	1	0	5	1	
1:4	1	10	8	10	6	6	4	16	10	
1:5	1	14	10	9	6	1	2	9	3	
1:6		6	6	7	6	3	1	10	4	Excluded no CFU
2:1	5	30	21	18	12	5	0	17	5	
2:2	7	136	58	24	4	3	0	7	3	
2:3	8	33	29	21	9	11	5	25	16	
2:4	12	27	24	22	15	4	1	20	5	
2:5		33	29	26	15	11	1	27	12	Excluded no CFU
2:6	7	34	28	21	18	9	1	28	10	
3:1	6	19	16	20	12	5	0	17	5	
3:2	17	700	224	53	30	10	6	46	16	
3:3	13	36	20	21	17	8	2	27	10	
3:4	13	29	24	24	18	8	1	27	9	
3:5	16	98	48	33	20	7	2	29	9	
3:6	3	98	55	27	20	9	4	33	13	
4:1	4	16	12	9	6	8	3	17	11	
4:2	2	22	16	8	5	6	2	13	8	
4:3	2	5	5	11	7	0	3	10	3	
4:4	2	7	6	10	2	2	1	5	3	
4:5	1	24	12	5	1	3	2	6	5	
4:6	3	12	9	8	1	3	2	6	5	
5:1	8	487	186	58	28	15	3	46	18	
5:2	1	102	46	11	4	4	1	9	5	
5:3	0	14	6	6	4	4	0	8	4	
5:4	7	30	11	22	13	11	4	28	15	
5:5	4	117	28	7	11	2	3	16	5	
5:6	13	23	16	24	18	2	1	21	3	
6:1	7	26	19	28	13	9	2	24	11	
6:2	8	318	78	18	12	7	4	23	11	
6:3	0	177	39	1	6	3	0	9	3	
6:4	2	5,726	1,984	468	98	22	2	122	24	Diathermy disturbances excluded in regressions
6:5	2	53	22	7	7	1	1	9	2	

6:6	10	41	20	26	17	7	2	26	9	
7:1	8	16	15	14	11	7	1	19	8	
7:2	9	166	85	33	13	7	3	23	10	
7:3	12	28	22	31	12	7	5	24	12	
7:4	23	18	15	13	13	5	5	23	10	
7:5	11	29	24	22	10	6	0	16	6	
7:6	8	25	15	20	17	9	3	29	12	
8:1	7	30	21	29	16	3	2	21	5	
8:2	15	87	45	34	16	14	2	32	16	
8:3	13	25	14	21	6	9	0	15	9	
8:4	10	16	13	17	8	9	1	18	10	
8:5	9	35	27	31	21	5	2	28	7	
8:6	24	780	283	97	39	17	9	65	26	
9:1	7	156	41	27	13	9	2	24	11	
9:2	15	22	13	15	11	2	2	15	4	
9:3	16	78	34	10	13	5	3	21	8	
9:4	18	68	36	19	11	9	1	21	10	
9:5	23	353	64	23	18	11	0	29	11	
9:6		102	31	26	20	13	5	38	18	Excluded no CFU
10:1	13	19	15	23	8	6	6	20	12	
10:2	11	95	40	14	5	8	4	17	12	
10:3	6	263	149	50	15	8	1	24	9	
10:4	8	25	17	11	13	7	1	21	8	
10:5	8	1,749	568	165	37	17	0	54	17	Diathermy disturbances , excluded in regressions
10:6	7	180	31	9	4	7	2	13	9	
11:1	34	65	46	31	15	10	0	25	10	
11:2	18	29	24	27	14	14	3	31	17	
11:3	31	342	224	110	50	29	2	81	31	
11:4	41	35	24	28	14	7	5	26	12	
11:5	23	38	29	31	15	14	4	33	18	
11:6	43	26	21	27	15	15	5	35	20	
12:1	6	14	13	7	11	2	2	15	4	
12:2	5	41	19	16	14	6	1	21	7	
12:3	3	147	76	32	5	3	1	9	4	
12:4	4	726	255	48	14	7	2	23	9	
12:5	8	459	234	79	19	9	2	30	11	
12:6	6	22	18	10	6	6	1	13	7	
13:1	9	21	16	16	9	5	0	14	5	

13:2	4	22	16	9	9	9	1	19	10	
13:3	11	148	39	12	9	6	0	15	6	
13:4	4	27	22	23	14	5	2	21	7	
13:5	4	14	10	17	7	1	0	8	1	
13:6	10	32	17	18	13	11	3	27	14	
14:1	4	16	14	8	11	7	1	19	8	
14:2		13	10	7	2	3	2	7	5	Excluded no CFU
14:3		17	14	7	6	2	0	8	2	Excluded no CFU
14:4	1	13	6	12	10	4	0	14	4	
14:5		21	8	8	4	5	1	10	6	Excluded no CFU
14:6	1	11	7	6	2	1	1	4	2	
15:1	2	10	8	7	5	2	1	8	3	
15:2	1	5	5	4	2	0	0	2	0	
15:3	5	20	15	9	6	5	4	15	9	
15:4	0	54	43	18	4	6	0	10	6	
15:5		11	10	3	3	6	1	10	7	Excluded no CFU
15:6	2	7	4	3	5	2	1	8	3	
16:1	4	26	17	18	14	10	2	26	12	
16:2	1	18	16	12	6	6	1	13	7	
16:3	1	40	29	18	14	9	2	25	11	
16:4	7	15	14	20	11	8	0	19	8	
16:5	2	26	25	25	12	10	2	24	12	
16:6	3	33	21	28	14	6	1	21	7	
17:1	3	60	39	20	6	6	2	14	8	
17:2	4	18	10	15	6	5	0	11	5	
17:3	1	16	11	8	5	2	1	8	3	
17:4	2	7	5	6	0	3	2	5	5	
17:5	2	8	7	14	6	2	0	8	2	
17:6	4	17	10	12	6	4	0	10	4	
18:1	2	314	79	21	4	5	1	10	6	
18:2	2	119	36	23	16	7	0	23	7	
18:3	0	526	276	110	41	28	4	73	32	Diathermy disturbances , excluded in regressions
18:4	0	733	455	225	79	33	4	116	37	Diathermy disturbances , excluded in regressions
18:5	1	34	25	17	12	3	1	16	4	
18:6	3	21	17	24	12	2	2	16	4	
